# A Deep Learning-Based Correction for Scanning Radius Errors in Circular-Scan Photoacoustic Tomography

**DOI:** 10.3390/jimaging12030097

**Published:** 2026-02-25

**Authors:** Jie Yin, Yingjie Feng, Junjun He, Min Xie, Chao Tao

**Affiliations:** 1 School of Electrical and Control Engineering, Nanjing Polytechnic Institute, Nanjing 210048, China; xm@njpi.edu.cn; 2 MOE Key Laboratory of Modern Acoustics, Department of Physics, Collaborative Innovation Center of Advanced Microstructures, Nanjing University, Nanjing 210023, China; fyj010825@163.com (Y.F.); taochao@nju.edu.cn (C.T.); 3 School of Intelligent Manufacturing, Nanjing Polytechnic Institute, Nanjing 210048, China; hejunjun@njpi.edu.cn

**Keywords:** photoacoustic tomography, scanning radius error, deep learning, ResNet, artifact correction

## Abstract

Circular-Scan photoacoustic tomography (PAT) can provide high-resolution images of optical absorption, but its analytical reconstructions, such as delay-and-sum (DAS), are highly sensitive to scanning radius (SR) inaccuracies, which cause severe geometric distortions and artifacts. In this work, we propose a deep learning framework, termed smooth deconvolution ResNet (SD-ResNet), to correct DAS reconstruction degradation induced by SR errors. SD-ResNet uses an ImageNet-pretrained ResNet-50 encoder and a lightweight deconvolutional decoder with additional smoothing convolutions to suppress checkerboard artifacts and restore fine structural details. A paired training dataset is generated using k-Wave simulations driven by human thoracic computed tomography (CT) slices: for each phantom, radiofrequency data are simulated once, and DAS images reconstructed with the true SR serve as ground truth, whereas images reconstructed with biased SR values serve as inputs. This design provides structurally diverse training samples and enhances generalization. In silico experiments show that SD-ResNet effectively recovers image quality across a range of SR deviations. Phantom experiments with polyethylene microspheres further confirm that the proposed method can substantially reduce artifacts and recover correct source shapes under practical SR mismatches, offering a robust tool for SR-error-resilient PAT imaging.

## 1. Introduction

Photoacoustic tomography (PAT) is a non-invasive hybrid imaging technique that combines optical excitation with ultrasonic detection to visualize the distribution of optical absorption in biological tissues [1,2,3,4,5]. PAT uses a non-focused pulsed laser beam to illuminate the imaging region and generate photoacoustic signals, which are then received by an array of transducers placed around the biological tissue. Reconstruction algorithms are used to recover the initial pressure distribution, which is directly related to local optical absorption [6,7]. In particular, by performing a 360° scan with a single ultrasound transducer or a transducer array, ring-shaped PAT offers full-view angle, and enables distortion-free, comprehensive visualization of internal physiological structures, making it widely used in various preclinical and clinical imaging applications [8,9,10].

Several algorithms have been developed to reconstruct PAT images utilizing the recorded radiofrequency (RF) signals, such as delay-and-sum (DAS) [11], back-projection (BP) method [12,13], time-reversal (TR) [14,15] and model-based (MB) methods [16,17]. DAS, BP, and TR are analytical methods that are computationally efficient; however, they generally do not provide accurate quantitative estimates of the imaging region [18,19]. The iterative approaches can achieve accurate quantitative estimation, but they are computationally expensive. In practical applications, especially in real-time and high-speed imaging systems, the primary reconstruction algorithms still rely on DAS and BP [20].

However, the quality of DAS/BP-based PAT reconstruction is highly sensitive to the accuracy of the system’s geometric parameters, especially the scanning radius (SR) in circular scan configurations. In mechanically scanned PAT systems that employ a single or multiple single-element transducers, SR inaccuracies are ubiquitous due to mechanical tolerances and positioning errors of each transducer [21]. Even in fixed full-ring PAT systems, fabrication and assembly tolerances introduce an effective radius that deviates from the assumed ideal SR, thereby degrading reconstruction quality [22]. The DAS/BP assume a precise known SR, and even a slight deviation of the SR from its true value can lead to severe image distortion and artifacts. In practice, miscalibration or experimental uncertainties in SR introduce geometric mismatches that manifest as blurring, geometric warping, and structural degradation in the reconstructed images. It is worth noting that accurately determining the SR in experimental settings sometimes can be a tedious and challenging task [21]. In practical scenarios, an initial estimate of the SR is typically used to generate a reconstructed image (e.g., via the DAS algorithm). The SR is then manually adjusted to achieve the most visually satisfactory image.

In recent years, with the rapid advances in deep learning (DL) techniques, DL has played an increasingly important role in PAT reconstruction tasks, including reconstruction from sparse-view measurements [23,24], convolutional neural network (CNN)-based bandwidth enhancement and sinogram super-resolution for limited-data acquisition [25], multiscale super-resolution in optical-resolution PAT [26], and human volumetric photoacoustic imaging in vivo [27]. Meanwhile, deep learning networks based on U-Net and DU-Net have also been employed to correct image degradation in PAT caused by inaccurate SR [28,29]. Ref. [28] mainly targets multiple single-element configurations and addresses per-transducer radius variations via a calibration-related workflow. Ref. [29] specifically processes three types of images, which first requires image classification, followed by further optimization.

This work aims to complement existing SR-correction methods by targeting a different yet common practical scenario—global SR deviation in circular-scan PAT (such as mechanically rotated single-element systems). In such settings, the effective SR may vary across sessions and repeated calibration or manual SR tuning can be inconvenient; therefore, an automatic one-shot post-reconstruction correction can improve usability and consistency. We propose a DL framework termed smooth deconvolution ResNet (SD-ResNet) to improve PAT reconstruction quality under SR errors. The SD-ResNet is built upon a ResNet-50 encoder that extracts multi-scale feature representations, coupled with a decoder designed to suppress checkerboard artifacts and faithfully restore fine structural details. Our network takes as input a distorted PAT image reconstructed with an incorrect SR and predicts a corrected image that more closely resembles the true source. To train the model, we generated a paired dataset using k-Wave simulations based on human computed tomography (CT) images: the RF ultrasound data were simulated for each phantom, and DAS reconstruction was performed using both the true SR (to produce the ground-truth image) and an erroneous SR (to produce the network input). This training strategy enables the model to learn a direct mapping from artifact-contaminated reconstructions to artifact-free images. Unlike previous studies that rely on simple geometric phantoms or limited image types, our approach uses real human thoracic CT images as the source for photoacoustic simulation. These realistic anatomical structures provide a diverse and representative dataset that enriches the structural variability of the training samples. This design choice enhances the generalization capability of the network, enabling it to perform robust corrections even on previously unseen image types or structural patterns.

We validate the proposed SD-ResNet on both simulated and phantom experiments under various SR mismatch conditions. Quantitative results demonstrate that our method effectively corrects the image quality degradation in DAS reconstructions caused by SR errors. Visually, the SD-ResNet reconstructions exhibit clearer structures and fewer distortions or artifacts even when the initial DAS images are severely warped by SR errors. Notably, the model also generalizes well to objects and image types not seen in training (e.g., phantoms not derived from CT scans), highlighting its robustness. In summary, the introduced SD-ResNet offers an effective and generalizable solution to mitigate SR-induced artifacts in PAT, improving image fidelity while reducing reliance on repeated calibration or manual SR tuning in scenarios where they are inconvenient or unstable.

## 2. Materials and Methods

**Figure 1:**
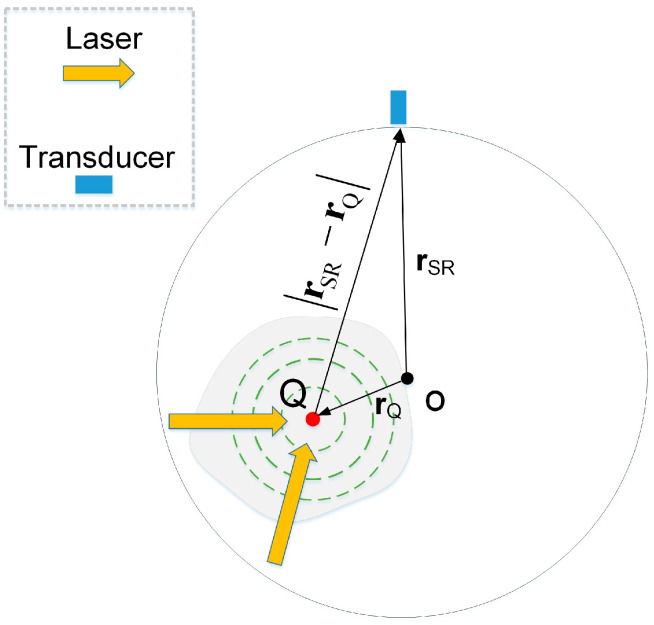
Schematic illustration of the PAT acquisition geometry. Point **O** denotes the center of the circular PAT scanning trajectory, the red point **Q** represents the photoacoustic source, **r**_SR_ is the SR vector, and **r**_Q_ is the vector from point **O** to point **Q**.

The PAT image reconstruction aims to determine the initial pressure rise from a series of collected acoustic signals. A nanosecond laser pulse irradiates the tissue, causing optical absorbers to experience thermoelastic expansion and generate broadband ultrasound waves, i.e., photoacoustic (PA) waves, as schematically shown in Figure 1.

The pressure *p*(**r**, *t*) at position **r** and time *t* in an acoustically homogeneous medium in response to a laser pulse *I*(*t*) obeys the following equation [12]:

(1) $$\nabla^{2}p(r,t)-\frac{1}{c^{2}}\frac{\partial^{2}}{\partial t^{2}}p(r,t)=-\frac{\beta }{C_{p}}\frac{\partial }{\partial t}A(r)I(t)$$

where *C_p_* is the specific heat, *A*(**r**) is the optical energy deposition per unit volume, *β* is the isobaric volume expansion coefficient, and *c* is the speed of sound. A transducer array is employed to acquire the PA signals. After transducers capture the PA signals, the initial pressure at **r** can be reconstructed via the DAS method:

(2) $$p_{0}(r)=\sum_{i=1}^{n}p_{i}(t-t_{i}),$$

where *p_i_*(*t*) is the PA signal recorded by the i-th transducer, the acoustic time-of-flight from the photoacoustic source at point **Q** to the transducer is given by $t_{i}=\left|r_{\mathrm{SR}}-r_{Q}\right|/c$. It can be seen that an accurate measurement of the SR is crucial for ensuring high-quality image reconstruction.

### 2.1. Training Dataset Preparation

To ensure that our synthetic phantoms encompass the diversity of structures encountered in practical PAT, we collected an open-access human thoracic 3D CT dataset [30] and used it as the photoacoustic source for simulation. From this dataset, we randomly selected 20 slices from each of 24 CT cases, yielding 480 thoracic CT slices in total. In this work, a “virtual phantom” refers to a 2D initial-pressure map constructed from one CT slice after preprocessing, which is used as PA source in the k-Wave simulation.

The detailed procedures are as follows: The numerical forward simulation of photoacoustic signal generation was performed using the k-Wave toolbox in a homogeneous acoustic medium. Human thoracic CT slices (512 × 512) were normalized, zero-padded to 768 × 768 and used as the initial pressure distribution *p*_0_(**r**). The computational grid had spatial steps of *dx* = *dy* = 40 µm, and the temporal sampling rate was 50 MHz, corresponding to 4500 sampling points. A circular detection array with 256 evenly spaced sensors was positioned around the imaging domain at a radius of 24.8 mm. Each transducer was modeled with a center frequency of 5 MHz and a bandwidth of 70%. The time-resolved acoustic pressure *p_i_*(*t*) was solved using GPU-accelerated kspaceFirstOrder2D, assuming constant speed of sound *c* = 1500 m/s, density *ρ* = 1000 kg/m^3^, and an acoustic absorption coefficient of 0.5 dB/(MHz·cm). The simulated time-series signals were stored as three-dimensional arrays, forming the RF datasets used for subsequent image reconstruction and network training.

**Figure 2:**
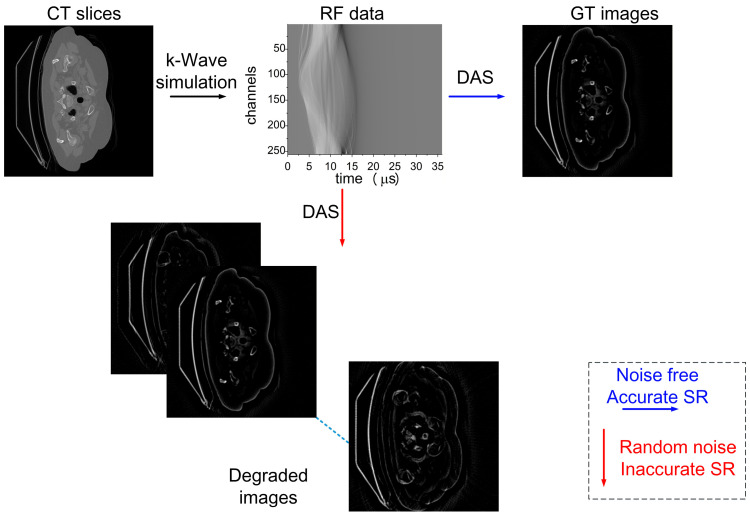
Training data generation workflow diagram. CT slices are used as photoacoustic sources to generate RF data. Noise-free reconstructions with the accurate SR serve as GT images, while noisy reconstructions with biased SR values form defective images. The two are paired for supervised training.

Subsequently, the RF signals were reconstructed using the DAS algorithm. A reconstruction radius of 24.8 mm was first adopted under noise-free conditions to obtain reference images for each RF dataset, which were regarded as the ground truth (GT). To emulate radius calibration errors, the reconstruction radius was then perturbed within a ±3.3% range—corresponding to radii from 23.8 mm to 25.8 mm in 0.20 mm increments—while keeping the acoustic velocity fixed at 1500 m/s. In addition, −30 dB random white noise was added to the RF signals. For each RF dataset, a total of 11 degraded images were reconstructed and paired with the corresponding GT image, forming a supervised training dataset for SR error correction. An overview of the training data generation pipeline and sample artifact–GT pairs is illustrated in Figure 2.

### 2.2. Network Architecture

**Figure 3:**
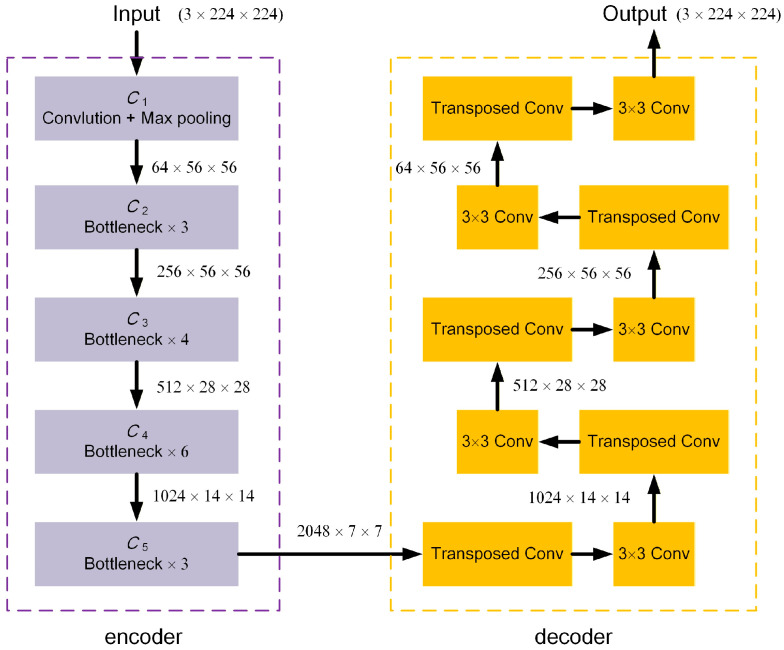
The framework of the proposed SD-ResNet. The left purple dashed box corresponds to the encoder, built upon an ImageNet-pretrained ResNet-50 backbone, while the right yellow dashed box corresponds to the decoder, consisting of transposed-convolution blocks and 3 × 3 convolution blocks.

In this work, we propose a smooth deconvolution ResNet (SD-ResNet), which adopts a ResNet-50 backbone with ImageNet pre-training as the encoder and employs a lightweight anti-checkerboard deconvolutional decoder for artifact correction. Given a 224 × 224 input patch (3 channels formed from the normalized grayscale), the encoder outputs multi-scale features {*C*_2_, *C*_3_, *C*_4_, *C*_5_} = {256 × 56 × 56, 512 × 28 × 28, 1024 × 14 × 14, 2048 × 7 × 7}. In this study, only the deepest feature map *C*_5_ is forwarded to the decoder, because SR-mismatch artifacts are mainly global and spatially correlated distortions, which are better captured by high-level features with a large receptive field. Using only *C*_5_ also keeps the decoder compact and efficient, avoiding the additional parameters and memory introduced by multi-scale skip connections (e.g., *C*_2_–*C*_4_), which did not provide consistent gains for this specific correction task in our preliminary tests. The decoder performs five-stage progressive upsampling using transposed convolutions (kernel size 4, stride 2), expanding the spatial resolution from 7 → 14 → 28 → 56 → 112 → 224. To explicitly suppress the checkerboard artifacts commonly introduced by conventional deconvolution, each upsampling stage is followed by a 3 × 3 convolutional smoothing layer with batch normalization and ReLU activation. This structure homogenizes kernel overlap patterns and stabilizes the upsampling process without relying on attention or multi-scale skip-fusion. This architecture provides a balance between representational capacity and computational efficiency. The pretrained ResNet-50 encoder contributes feature abstraction, while the anti-checkerboard deconvolutional decoder enables structural restoration with reduced grid-like artifacts and improved local smoothness. The overall framework is illustrated in Figure 3.

### 2.3. Training Strategy

The proposed SD-ResNet framework was trained using paired artifacts–GT images produced with the method in Section 2.1. Before training, the dataset was randomly divided into training, validation, and testing subsets using a 7:2:1 ratio. To improve data consistency, both artifact and GT patches were center-cropped from 512 × 512 to 400 × 400 pixels and further randomly sampled into 224 × 224 patches during training. A pairwise intensity rescaling procedure was applied before feeding data into the network: the 0.5–99.5 percentile range of each GT patch defined a shared normalization interval for the artifact–GT pair, ensuring a consistent dynamic range across samples. After this normalization, the DAS-reconstructed grayscale input patch was replicated into three identical channels to match the 3-channel interface of the ImageNet-pretrained ResNet-50 encoder (i.e., *I*_3ch_(x,y) = [*I*_norm_(x,y), *I*_norm_ (x,y), *I*_norm_ (x,y)]). The three channels are identical and do not represent different physical quantities. Standard geometric augmentations (random horizontal and vertical flips) were used.

We used ImageNet-pretrained ResNet-50 as the encoder and initialized all decoder parameters randomly. All batch normalization layers in the encoder were forced to operate in evaluation mode to avoid instability caused by small batch sizes. The network was optimized using AdamW with decoupled weight decay. A two-stage learning rate schedule was adopted: a 5-epoch linear warm-up, followed by cosine-annealing decay until the final epoch. Additionally, an exponential moving average (EMA) of model weights was maintained with a decay of 0.992, and EMA weights were used for validation inference. We use *L*_1_ loss as the loss function for training:

(3) $$L=\frac{1}{N}\sum_{i=1}^{N}\sqrt{{\left(x_{i}-y_{i}\right)}^{2}},$$

A gated checkpointing strategy was used during training. The best model (raw weights and EMA weights) was saved only after epoch 21 to ensure that early unstable fluctuations did not dominate model selection. Improvement was determined by relative validation loss reduction, and a separate last-epoch checkpoint was always stored.

In most experiments, the best validation performance was achieved at about 1000 epochs. We set the maximum number of epochs to 2000 only as a conservative upper bound to ensure sufficient optimization in rare cases where convergence is slower. Training was terminated early if the validation loss did not improve for more than 20 consecutive epochs.

## 3. Results

### 3.1. In Silico Experiments

We first evaluated SD-ResNet’s performance under controlled simulation conditions. In this work, each virtual phantom is a 2D initial-pressure map constructed from one thoracic CT slice as described in Section 2.1. We generated 480 virtual phantoms from 480 CT slices. The phantom dataset was split into 336/96/48 phantoms for training/validation/testing. All models were trained and tested on an Ubuntu workstation (Intel Core i7 CPU (Intel Corporation, Santa Clara, CA, USA), 24 GB RAM (Samsung Electronics Co., Ltd., Suwon-si, Gyeonggi-do, Korea), NVIDIA RTX 4070 Ti GPU (NVIDIA Corporation, Santa Clara, CA, USA)).

We trained the network following the procedure described in Section 2.2, and the best-performing model was obtained after approximately 900 epochs. To provide representative examples, we first selected two test set slices with distinct structural characteristics.

**Figure 4:**
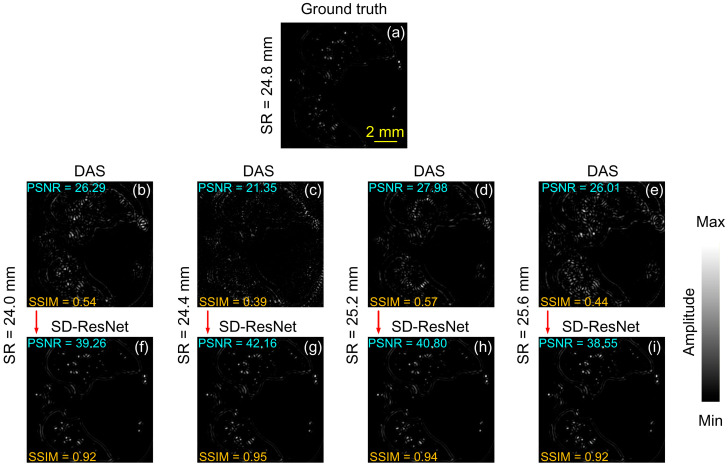
Reconstruction and correction for Slice 1. (**a**) GT image reconstructed by DAS under noise-free conditions with the accurate SR = 24.8 mm. (**b**–**e**) DAS reconstructions obtained under noisy conditions with SR = 24.0, 24.4, 25.2, and 25.6 mm, respectively. (**f**–**i**) Corrected results produced by feeding (**b**–**e**) into the proposed SD-ResNet. The red arrow indicates the output result of SD-ResNet.

Slice 1 contains predominantly point-like, circular, and elliptical absorbers, as illustrated in Figure 4. The original images have a resolution of 512 × 512 pixels. For clearer visualization of fine details, we cropped a central 300 × 300 region from each image for display. Each reconstructed image is normalized by its highest pixel value. Figure 4a contains the GT image for the phantom. Figure 4b–e show the DAS reconstruction results obtained using different reconstruction SR, specifically 24.0, 24.4, 25.2, and 25.6 mm, respectively. Figure 4f–i present the corresponding reconstruction results after correction by the proposed SD-ResNet. The computed values of Peak Signal-to-Noise Ratio (PSNR) and Structural Similarity Index Measure (SSIM) are inserted in each image.

PSNR is defined as:

(4) $$\mathrm{PSNR}(x,y)=10log_{10}(\frac{L^{2}}{\mathrm{MSE}(x,y)}),$$

where *x* and *y* denote the predicted image and the GT respectively. *L* represents the maximum possible pixel intensity (set to 1 for normalized images), and MSE(*x*, *y*) is the mean squared error between the two images. Higher PSNR values indicate lower reconstruction error and improved fidelity relative to the GT image.

SSIM is defined as:

(5) $$\mathrm{SSIM}(x,y)=\frac{\left(2\mu_{x}\mu_{y}+C_{1})(2\sigma_{xy}+C_{2}\right)}{\left(\mu_{x}^{2}+\mu_{y}^{2}+C_{1})(\sigma_{x}^{2}+\sigma_{y}^{2}+C_{2}\right)},$$

where *x* and *y* denote the predicted image and the GT respectively. *µ_x_* and *µ_y_* represent their mean pixel intensities, *σ_x_* and *σ_y_* their standard deviations, and *σ_xy_* the covariance between *x* and *y*. *C*_1_ = 0.01 and *C*_2_ = 0.03 are small stabilizing constants. SSIM assesses perceptual and structural fidelity, with a theoretical range of −1 to 1 (where 1 denotes identical structure, 0 no correlation, and negative values an inverse relationship), but in most imaging contexts falls between 0 and 1.

As shown in the figures, deviations in the SR lead to a substantial degradation in DAS image quality. Severe artifacts and geometric distortions appear across all SR-mismatched reconstructions, and in some cases (e.g., Figure 4c), the coherent structures become almost unrecognizable. In contrast, the proposed SD-ResNet successfully restores the major anatomical structures and fine details, producing visibly improved reconstructions. The corresponding PSNR and SSIM values are consistent with these visual impressions.

**Figure 5:**
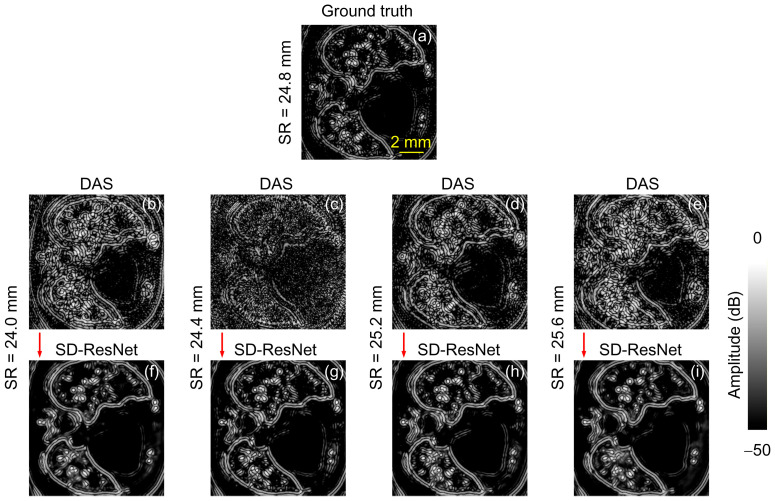
Log-scale (dB-compressed) visualizations corresponding to Figure 4. (**a**) GT image reconstructed by DAS under noise-free conditions with the accurate SR = 24.8 mm. (**b**–**e**) DAS reconstructions obtained under noisy conditions with SR = 24.0, 24.4, 25.2, and 25.6 mm, respectively. (**f**–**i**) Corrected results produced by feeding (**b**–**e**) into the proposed SD-ResNet. The red arrow indicates the output result of SD-ResNet.

In addition to the linear-scale results in Figure 4, we provide the corresponding log-scale (dB-compressed) visualizations in Figure 5 to facilitate qualitative assessment of low-amplitude structures and residual artifacts. Specifically, the image is first normalized by its maximum value and then converted to a dB scale as:

(6) $$I_{dB}(x,y)=20\log_{10}(\frac{I(x,y)}{\max(I)+\varepsilon }),$$

where ϵ is a small constant to avoid log(0). The dB images are displayed with a fixed dynamic range of 50 dB (from −50 dB to 0 dB). These log-scale views make subtle artifacts and weak features more discernible and provide complementary visual evidence of the artifact suppression achieved by SD-ResNet.

**Figure 6:**
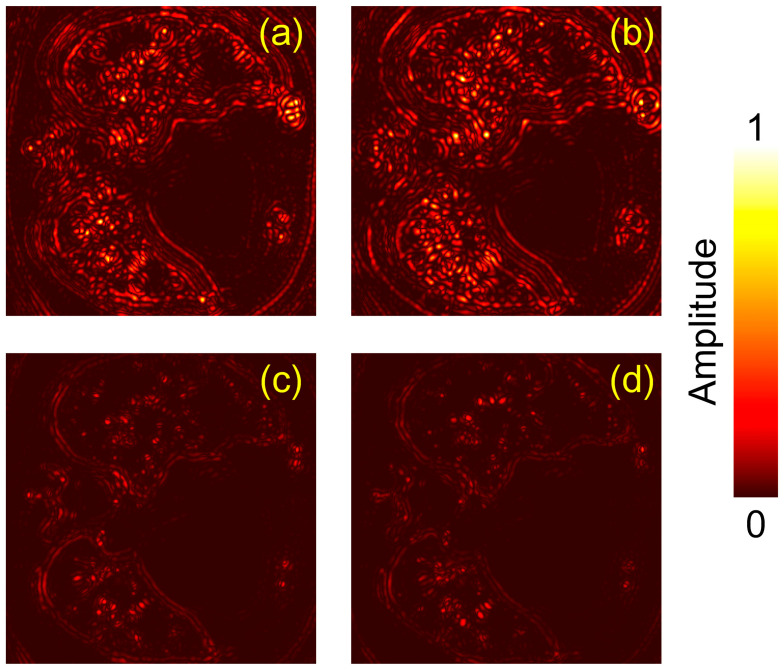
Absolute error maps (linear scale) for Slice 1 relative to the GT. (**a**) Absolute error map between Figure 4a,b. (**b**) Absolute error map between Figure 4a,e. (**c**) Absolute error map between Figure 4a,f. (**d**) Absolute error map between Figure 4a,i.

To further quantify and localize the residual distortions, we additionally visualize pixel-wise absolute error maps with respect to the GT. Specifically, the absolute error is computed as $E(r)=|I(r)-I_{GT}(r)|$, where I(r) is the linear pressure map, and $I_{GT}(r)$ is the GT image. All images are normalized using the same scaling for fair comparison. The resulting error maps for the DAS reconstructions and the corresponding SD-ResNet outputs are shown in Figure 6.

In these absolute error maps, brighter (higher) values indicate larger deviations from the GT, corresponding to more severe residual distortions and thus poorer reconstruction fidelity. Conversely, lower error magnitudes suggest closer agreement with the GT and improved reconstruction quality.

**Figure 7:**
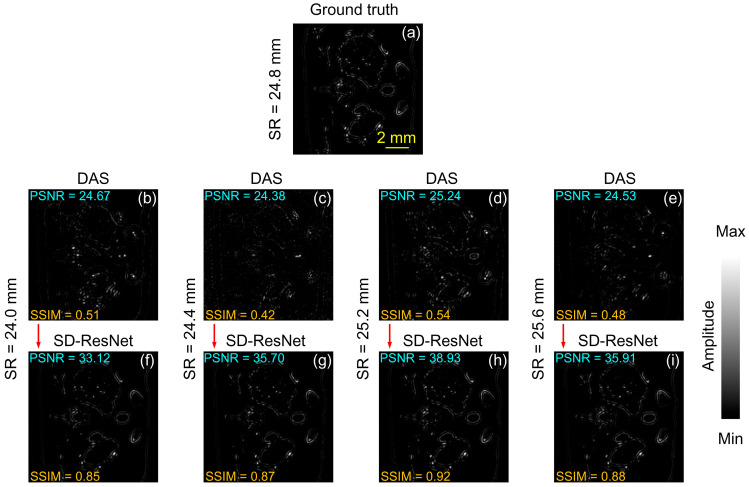
Reconstruction and correction for Slice 2. (**a**) GT image reconstructed by DAS under noise-free conditions with the accurate SR = 24.8 mm. (**b**–**e**) DAS reconstructions obtained under noisy conditions with SR = 24.0, 24.4, 25.2, and 25.6 mm, respectively. (**f**–**i**) Corrected results produced by feeding (**b**–**e**) into the proposed SD-ResNet. The red arrow indicates the output result of SD-ResNet.

Unlike Slice 1, the absorbers in Slice 2 predominantly exhibit irregular curvilinear shapes, as shown in Figure 7. The image’s SR selection, visualization procedure, and metric computation are identical to those used in Figure 4. It can be observed that, despite the distinct structural characteristics of the absorbers, SD-ResNet consistently improves the quality of photoacoustic tomographic reconstructions across both cases.

**Figure 8:**
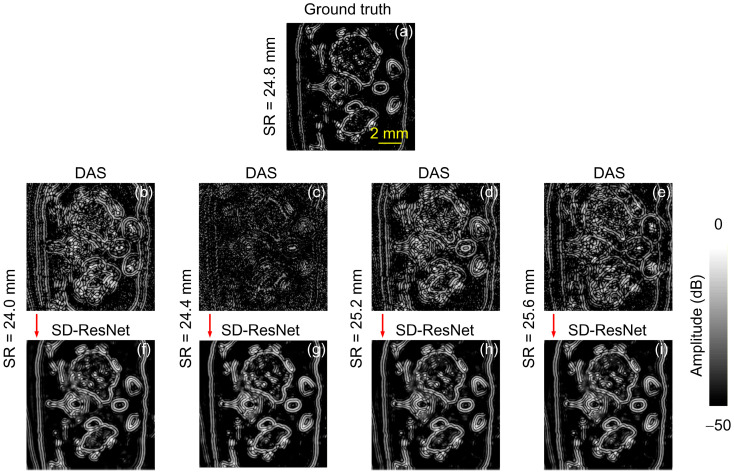
Log-scale (dB-compressed) visualizations corresponding to Figure 7. (**a**) GT image reconstructed by DAS under noise-free conditions with the accurate SR = 24.8 mm. (**b**–**e**) DAS reconstructions obtained under noisy conditions with SR = 24.0, 24.4, 25.2, and 25.6 mm, respectively. (**f**–**i**) Corrected results produced by feeding (**b**–**e**) into the proposed SD-ResNet. The red arrow indicates the output result of SD-ResNet.

We also provide the corresponding log-scale (dB-compressed) visualizations in Figure 8.

The log-scale (dB-compressed) visualizations support the same qualitative conclusions as the linear-scale images. They provide a clearer view of low-amplitude structures and residual artifacts, further confirming that SD-ResNet reduces SR-mismatch–induced artifacts while preserving the main structural features.

**Figure 9:**
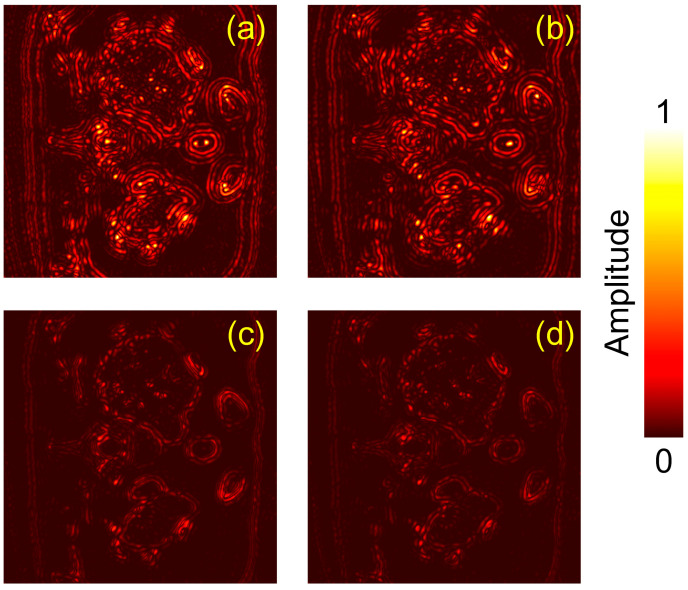
Absolute error maps (linear scale) for Slice 2 relative to the GT. (**a**) Absolute error map between Figure 7a,b. (**b**) Absolute error map between Figure 7a,e. (**c**) Absolute error map between Figure 7a,f. (**d**) Absolute error map between Figure 7a,i.

Using the same visualization procedure as in Figure 6, we also generated the corresponding error maps for Slice 2, as shown in Figure 9.

The error maps (Figure 6 and Figure 9) indicate that the SD-ResNet–corrected reconstructions exhibit substantially smaller deviations from the GT than the corresponding DAS results.

To further evaluate the effectiveness of SD-ResNet, we performed quantitative analysis on all 528 reconstructed images in the test set (48 slices × 11 SR conditions). In addition to PSNR and SSIM, our quantitative evaluation also includes the mean squared error (MSE) and the Pearson correlation coefficient (PCC). MSE quantifies the average pixel-wise intensity deviation from the GT, with values ranging from 0 (perfect agreement) to 1 (maximal error across a fully normalized [0, 1] image). The definition of MSE is given in Equation (7):

(7) $$\mathrm{MSE}(x,y)=\frac{1}{N}\sum_{i=1}^{N}{\left(x_{i}-y_{i}\right)}^{2},$$

The PCC measures the linear relationship between reconstructed and GT images, also ranging from −1 to 1; values closer to 1 indicate stronger agreement in overall intensity patterns and contrast. The definition of PCC is given in Equation (8):

(8) $$\mathrm{PCC}(x,y)=\frac{\sum_{i=1}^{N}\left(x_{i}-\mu_{x})(y_{i}-\mu_{y}\right)}{\sqrt{\sum_{i=1}^{N}{\left(x_{i}-\mu_{x}\right)}^{2}}\sqrt{\sum_{i=1}^{N}{\left(y_{i}-\mu_{y}\right)}^{2}}}$$

In Equations (7) and (8), *N* denotes the total number of pixels, *x* and *y* denote the predicted image and the GT respectively, *µ_x_* and *µ_y_* represent their mean pixel intensities.

The results are summarized in Table 1.

As shown in Table 1, SD-ResNet achieves substantial improvements over DAS across all quantitative metrics, which are consistent with visual observations.

Across the tested SR range (23.8–25.8 mm), SD-ResNet shows consistent correction performance; larger deviations will be investigated in future work by extending the perturbation range during training.

**Figure 10:**
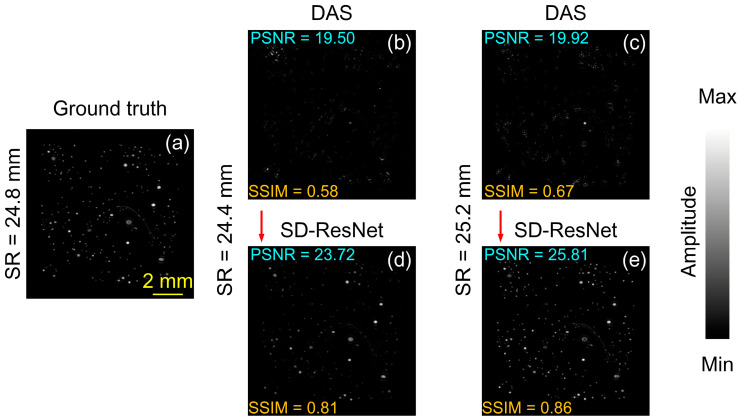
Reconstruction and correction for non-CT–based source 1. (**a**) GT image reconstructed by DAS under noise-free conditions with the accurate SR = 24.8 mm. (**b**,**c**) DAS reconstructions obtained under noisy conditions with SR = 24.4, and 25.2 mm, respectively. (**d**,**e**) Corrected results produced by feeding (**b**,**c**) into the proposed SD-ResNet. The red arrow indicates the output result of SD-ResNet.

To evaluate the generalization capability of the proposed method, we additionally selected two non-CT based source images for testing. One of them is an artificially generated phantom containing randomly distributed circular and curvilinear structures. The DAS reconstruction obtained under noise-free conditions using the accurate SR (24.8 mm) is shown in Figure 10a and is regarded as the GT. When the SR was incorrectly set to 24.4 mm and 25.2 mm, and −30 dB random white noise was added, the DAS reconstruction results are shown in Figure 10b,c. The corresponding images corrected by the proposed SD-ResNet are presented in Figure 10d and Figure 10e, respectively.

**Figure 11:**
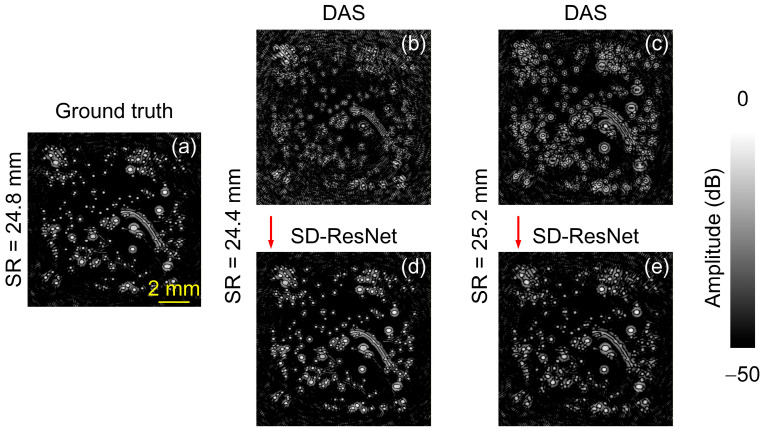
Log-scale (dB-compressed) visualizations corresponding to Figure 10. (**a**) GT image reconstructed by DAS under noise-free conditions with the accurate SR = 24.8 mm. (**b**,**c**) DAS reconstructions obtained under noisy conditions with SR = 24.4, and 25.2 mm, respectively. (**d**,**e**) Corrected results produced by feeding (**b**,**c**) into the proposed SD-ResNet. The red arrow indicates the output result of SD-ResNet.

The log-scale (dB-compressed) visualization corresponding to Figure 10 is shown in Figure 11.

**Figure 12:**
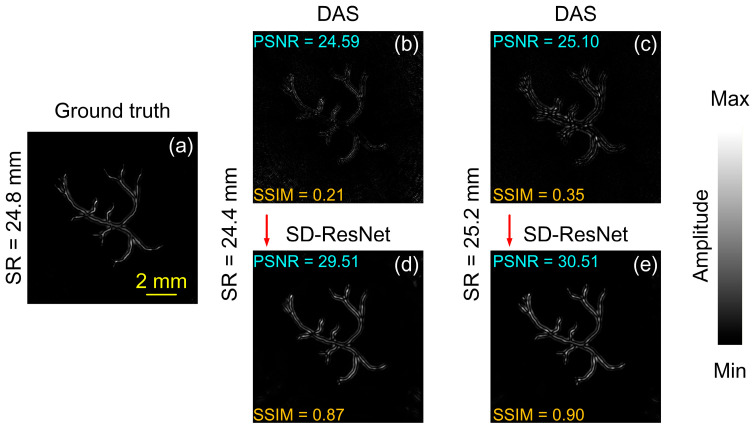
Reconstruction and correction for non-CT based source 2. (**a**) GT image reconstructed by DAS under noise-free conditions with the accurate SR = 24.8 mm. (**b**,**c**) DAS reconstructions obtained under noisy conditions with SR = 24.4 and 25.2 mm, respectively. (**d**,**e**) Corrected results produced by feeding (**b**,**c**) into the proposed SD-ResNet. The red arrow indicates the output result of SD-ResNet.

The other source image selected for testing is the synthetic vascular phantom provided in the k-Wave toolbox. The DAS reconstruction obtained under noise-free conditions using the accurate SR (24.8 mm) is shown in Figure 12a and is regarded as the GT. When the SR was incorrectly set to 24.4 mm and 25.2 mm, and −30 dB random white noise was added, the DAS reconstruction results are shown in Figure 12b,c. The corresponding images corrected by the proposed SD-ResNet are presented in Figure 12d and Figure 12e, respectively.

**Figure 13:**
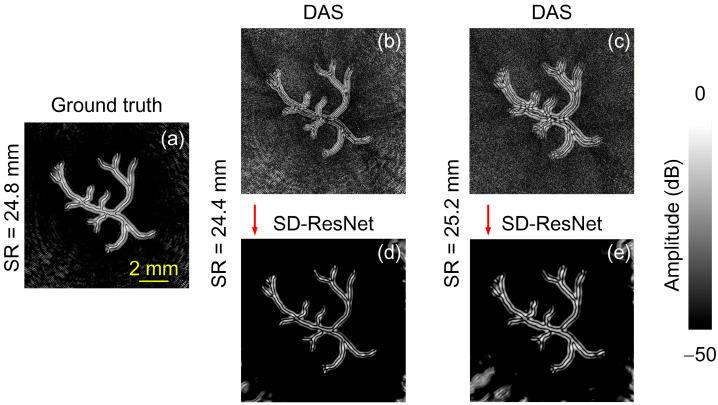
Log-scale (dB-compressed) visualizations corresponding to Figure 12. (**a**) GT image reconstructed by DAS under noise-free conditions with the accurate SR = 24.8 mm. (**b**,**c**) DAS reconstructions obtained under noisy conditions with SR = 24.4 and 25.2 mm, respectively. (**d**,**e**) Corrected results produced by feeding (**b**,**c**) into the proposed SD-ResNet. The red arrow indicates the output result of SD-ResNet.

The log-scale (dB-compressed) visualization corresponding to Figure 12 is shown in Figure 13.

From Figure 10, Figure 11, Figure 12 and Figure 13, it can be observed that although the selected source images differ substantially from the training data, the proposed network still provides significant improvements over the DAS reconstructions obtained with incorrect SR settings.

### 3.2. Phantom Experiments

**Figure 14:**
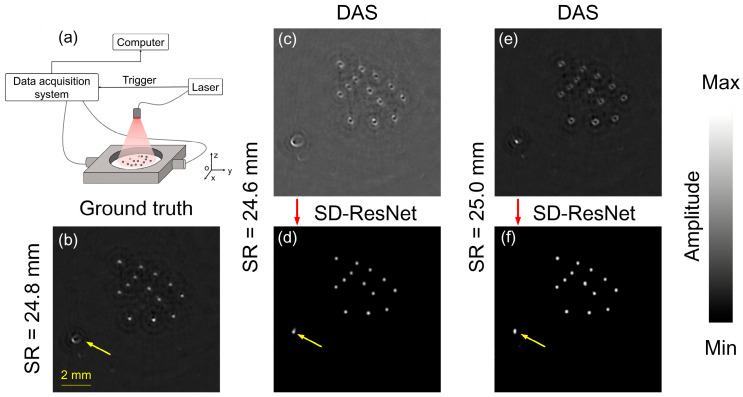
Phantom experiment setup. (**a**) Schematic diagram of the PAT system with a ring-shaped transducer array. (**b**) GT image reconstructed by DAS with the accurate SR = 24.8 mm. (**c**,**e**) DAS reconstructions obtained with SR = 24.6 and 25.0 mm, respectively. (**d**,**f**) Corrected results produced by feeding (**c**,**e**) into the proposed SD-ResNet. The red arrow indicates the output result of SD-ResNet. The yellow arrow indicates the off-center microsphere with residual distortion.

To further validate the performance of our method in practical settings, we conducted phantom experiments. The experimental setup is illustrated in Figure 14a. A Q-switched Nd:YAG laser (OPOTEK LLC, Carlsbad, CA, USA) served as the light source, generating pulses with a duration of 4.5 ns and a repetition rate of 10 Hz. A custom-designed self-focused concave ring-array transducer (Doppler Electronic Technologies, Guangzhou, China) was employed for signal acquisition. The array consisted of 256 elements with a ring diameter of 48 mm. The focus radius was approximately 9 mm. The element pitch was 0.6 mm (kerf 0.15 mm), and the element length was 10 mm. The transducer had a center frequency of 5 MHz with 70% (−6 dB) bandwidth. An ultrasonic acquisition system (Custom-designed, manufactured by Nanjing Genan Industrial Co., Ltd., Nanjing, China) was used to record the signals at a sampling rate of 50 MHz.

The optical absorbers used in the experiments consisted of 14 randomly distributed polyethylene microspheres (Zhuoyue Alloy, Dongguan, China), each with a diameter of 200 µm. The microspheres were embedded in 2% agarose (Foshan Krypai Chemical, Foshan, China), whose speed of sound was measured to be 1520 m/s. Using the actual SR of the transducer array (24.8 mm), the DAS reconstruction obtained under this SR setting is shown in Figure 14b. Subsequently, the SR was set to 24.6 mm and 25.0 mm, and the corresponding DAS reconstructions are shown in Figure 14c and Figure 14e, respectively. The reconstructions obtained using these incorrect SR were then fed into the proposed network, and the corrected results are presented in Figure 14d,f.

As shown in Figure 14, when the selected SR deviates from the true value during imaging, the DAS reconstructions exhibit pronounced artifacts and distortions. After feeding the DAS reconstructions into the SD-ResNet, the images are markedly improved: the artifacts disappear or are substantially reduced, the polyethylene spheres recover their true shapes and sizes, and the background noise within the imaging region is effectively suppressed. We also note that the reference (“GT”) image in the phantom experiment (Figure 14b) was obtained by manually tuning the reconstruction SR to achieve the visually best DAS result.

However, even under this carefully tuned condition, a microsphere located farther from the scanning center (indicated by the yellow arrow) still exhibits a non-ideal distorted appearance. This residual distortion is consistent with the spatially varying effective resolution of circular-scan DAS reconstruction, which arises primarily from the finite active aperture and receive directivity of practical detector elements. In particular, the angle-dependent receive sensitivity and spatial averaging lead to a position-dependent point-spread function, resulting tangential blurring for off-center targets. These effects cannot be fully compensated by a single global SR adjustment.

After SD-ResNet correction, the microsphere’s appearance becomes more regular and the surrounding artifacts are reduced. One possible explanation is that finite-aperture/directivity–related degradations can be coupled with, and co-exist alongside, SR-mismatch artifacts in the image domain, producing spatially correlated deformations with similar feature patterns. Since SD-ResNet learns a statistical mapping from artifact-contaminated DAS images to a reference reconstruction, it may also partially attenuate these coupled degradations through learned regularization. This observation suggests that the proposed method can serve as a complementary correction in scenarios where conventional manual calibration (i.e., global SR tuning) does not fully yield satisfactory reconstruction quality.

### 3.3. Computational Performance

Inference was carried out on the same desktop workstation described in Section 3.1, namely, an Intel Core i7 CPU with 24 GB RAM and an NVIDIA GeForce RTX 4070 Ti GPU. For inference on a single 224 × 224 image (batch size = 1), SD-ResNet requires 15 ms per forward pass with a peak GPU memory usage of 355 MB (DAS reconstruction excluded). These results indicate that the proposed framework enables near–real-time post-reconstruction correction on a modern GPU, supporting practical deployment in circular-scan PAT systems where fast artifact mitigation is desired.

## 4. Conclusions

In conclusion, this study presented a deep learning-based reconstruction method to counteract the detrimental effects of SR errors in circular-scan photoacoustic tomography. We developed the SD-ResNet architecture, which integrates a deep ResNet-50 encoder and a checkerboard-artifact-free decoder to directly learn the mapping from distorted PAT images to high-fidelity reconstructions. Trained on an extensive k-Wave–based simulated dataset derived from human thoracic CT images, the proposed network effectively recovers structural details that are lost or distorted by SR errors. As a result, our approach yields PAT images with substantially improved quality compared to the conventional DAS algorithm. Experiments on both synthetic data and phantom measurements demonstrate marked improvements in quantitative image quality metrics for the simulated cases, and visually cleaner, more geometrically faithful reconstructions with reduced artifacts in the experimental setting. The observed ability of SD-ResNet to generalize to non-CT-based phantoms indicates a promising level of robustness to unseen image types.

For sparse, point-like microsphere phantoms, SR mismatch can often be mitigated by conventional geometric calibration or manual SR tuning. In this work, the phantom experiment primarily serves to demonstrate the feasibility of the proposed correction on real measurements. The proposed SD-ResNet framework is particularly useful in scenarios where the effective SR is difficult to determine reliably or may vary across sessions, such as circular-scan systems using a rotating single-element transducer. In these systems, small SR deviations may be introduced by alignment errors or repositioning; consequently, repeated calibration or manual tuning can be inconvenient and operator-dependent. In contrast, SD-ResNet provides a one-shot post-reconstruction correction without additional calibration measurements, improving usability and robustness when calibration is unavailable, inconvenient, or unstable.

A limitation of the current experimental validation is that it was conducted on a microsphere phantom, which does not fully represent more challenging tissue-mimicking phantoms or in vivo conditions. Further validation on tissue-like phantoms and in vivo data will be pursued to better assess performance under realistic conditions.

Looking forward, there are several avenues for future work. One direction is to extend the proposed approach to three-dimensional PAT and in vivo imaging, which would further validate the method’s utility in preclinical or clinical contexts. Additionally, SR mismatch may coexist with acoustic heterogeneity in practice, which may reduce performance due to a distribution shift of artifact patterns. Future work will explore combined-perturbation datasets and joint/multi-stage training to improve robustness and potentially mitigate both SR and acoustic-parameter mismatches. Moreover, Monte Carlo–based optical modeling could be integrated to produce more realistic initial pressure distributions, enabling validation under strongly heterogeneous fluence conditions.

## Figures and Tables

**Table 1:** Test-Set Performance Comparing SD-ResNet with DAS. Blue values indicate an increase in the metric value relative to DAS, whereas red values indicate a decrease in the metric value relative to DAS.

	PSNR	SSIM	MSE	PCC
DAS	25.34	0.46	0.022	0.43
SD-ResNet	39.09 (↑54%)	0.92 (↑100%)	0.0026 (↓88%)	0.91 (↑110%)

## Data Availability

The raw data supporting the conclusions of this article will be made available by the authors on request.
